# Physical activity phenotypes from volume-based versus intensity-based wearable metrics: discordant classification and divergent metabolomic, body composition, and cardiac magnetic resonance associations

**DOI:** 10.3389/fdgth.2026.1982891

**Published:** 2026-10-26

**Authors:** Sarah Shi Sien Goh, Khung Keong Yeo, Jonathan Yap, Siew Ching Kong, Weng Khong Lim, Mark Kei Fong Wong, Eddie Yin Kwee Ng, Xiayan Shen, Alex Weixian Tan, Weiting Huang

**Affiliations:** 1 Yong Loo Lin School of Medicine, National University of Singapore, Singapore; 2 National Heart Centre Singapore, Singapore; 3 Duke-NUS Medical School, Singapore; 4 Nanyang Technological University, Singapore

**Keywords:** accelerometry, cardiometabolic risk, cluster analysis, digital health, metabolic equivalent of tasks, physical activity, validation studies, wearable devices

## Abstract

**Background:**

Consumer wearables report direct volume-based metrics (steps, distance, floors climbed) alongside device-derived metrics generated by manufacturer algorithms (activity-intensity minutes, energy expenditure). Both are widely used to characterize individuals into activity phenotypes, yet whether these representations yield concordant activity phenotypes and biological associations is unclear.

**Objective:**

To compare activity phenotypes derived from direct accelerometry with those derived from device-derived intensity metrics, and to evaluate each against metabolomic, body composition, and cardiac magnetic resonance (CMR) phenotypes.

**Methods:**

Using the SingHEART cohort, two hierarchical cluster analyses using Ward linkage yielded five phenotypes each for volume-based and intensity-based metrics. Agreement was quantified by the adjusted Rand index. For analyses, the two high-variability phenotypes were combined, giving four groups: sedentary, average movers, regular exercisers, and high-variability movers. Serum ¹H-NMR metabolomics was analyzed by PERMANOVA with Holm adjustment across pairwise contrasts. Body and CMR associations were assessed using linear regression with false discovery rate correction.

**Results:**

In total, 984 adults (mean age 45.6 years, 48.3% male) wore accelerometers for a mean of 7.1 valid days. The two classifications agreed poorly: adjusted Rand index was 0.18, with only 46.2% assigned identically. Volume-based phenotypes differed in metabolomic profiles (*P* < .001). Active participants (high-variability movers plus regular exercisers) differed from sedentary participants across 106 of 250 biomarkers (FDR *q* < 0.05), including higher HDL, lower VLDL, LDL and lower GlycA. Conversely, although intensity-based phenotypes showed a nominally significant omnibus metabolomic association (*P* = .003), no metabolomic difference was observed between active and sedentary groups defined by intensity-based phenotyping (*P* = .29). Across 16 body composition and CMR outcomes, volume-based phenotypes were associated with 11 and intensity-based phenotypes with 6, in divergent directions: volume-based high-variability movers showed lower body fat, higher skeletal muscle, and larger ventricular volumes and mass, whereas intensity-based high-variability movers showed higher body mass index and waist circumference.

**Conclusions:**

Volume-based metrics and intensity-based metrics assign the same individuals to discordant activity phenotypes. Volume-based phenotypes showed more consistent associations across metabolomic, body composition, and cardiac imaging measures. These findings suggest that the choice of wearable-derived metrics substantially influences digital activity phenotyping and its biological interpretation.

## Introduction

The rapid proliferation of wearable technology has transformed the landscape of physical activity research, giving clinicians unprecedented access to longitudinal activity data (1, 2). Besides providing information on one's daily step count, wearables also calculate derived metrics that estimate energy expenditure and categorize activity intensity using Metabolic Equivalent of Tasks (METs) thresholds (3). Notably, a significant amount of literature relies heavily on these intensity-based metrics like METs in predicting long-term outcomes like cardiometabolic disease, mortality, and other heart diseases (4–6).

While METs provide a standardized language for public health guidelines (7), derived metrics are based on proprietary algorithms, and relying on them could obscure the true relationship between physical activity and health outcomes. Validation studies have demonstrated that different wearable devices showed weak correlations in measuring physical activity based on METs (8, 9). Furthermore, systematic errors in heart rate monitoring for darker skin tones (10) and the misclassification of activity intensity in higher body mass index populations (11) have raised concerns regarding the equity and accuracy of derived metabolic metrics.

To assess the validity of activity algorithms for phenotyping, digital health metrics should be evaluated against objective biological measures. Circulating metabolomic profiling provides an in-depth assessment of metabolic health, capturing variations in lipoprotein subfractions, lipid concentrations, and inflammatory markers that may reflect differences in physical activity and fitness (12). Assessment of body composition also provides insight into changes in body fat and skeletal muscle mass associated with regular physical activity. Cardiac magnetic resonance (CMR) imaging is an established reference method for assessing cardiac structure and function, allowing evaluation of cardiovascular adaptations (13).

This study aims to compare two physical activity phenotyping strategies applied to a population-based cohort: one using direct accelerometry metrics (step counts, distance, floors climbed, and day-to-day variability) and the other using device-derived intensity metrics (activity intensity minutes and caloric expenditure). We evaluate the concordance between these two classification schemes and test their comparative validity against serum ¹H-NMR metabolomics, detailed body composition parameters, and CMR-derived cardiac phenotypes.

## Methods

### Study design and population

Participants were enrolled in the SingHEART study, a population-based cohort study conducted in Singapore designed to better understand the determinants of cardiovascular health among the healthy Asian population (14). Males and females aged 21–69 years were recruited for the study. As this is a study on healthy individuals, those with known cardiovascular diseases (such as coronary artery disease, peripheral arterial disease or a previous myocardial infarction), previous stroke or diabetes mellitus were excluded. A prior medical history of hypertension was permitted as long as patients were not on more than one anti-hypertensive medication.

Volunteers were recruited by print advertising to join the SingHEART study by the National Heart Centre Singapore (NHCS), which has received ethics approval from the SingHealth Centralized Institutional Review Board (ref: 2015/2601). Volunteers had to complete a written informed consent form before information was collected following the study's protocol.

Participants completed standardized questionnaires to collect information on their demographics, medical history, socioeconomic status and lifestyle factors. Standard clinical parameters such as height, weight, waist and hip circumferences were measured and used to calculate body mass index and waist-to-hip ratio. Laboratory investigations including a fasting lipid panel and blood glucose were also performed.

### Wearable device data collection

Physical activity data was collected using Fitbit Charge HR wrist-worn accelerometers (Fitbit Inc., San Francisco, CA), which participants wore for a monitoring period of up to 8 consecutive days. Participants were instructed to wear the Fitbit Charge HR continuously, 24 h a day, removing it only for charging. Devices recorded multiple volume-based daily activity metrics including step counts, distance walked (km), floors climbed, as well as derived metrics such as minutes in each activity intensity zone (sedentary, lightly active, fairly active, and very active), activity calories, and total calories burned.

### Derivation of summary physical activity metrics

For each participant, summary metrics were derived across all valid wear days to characterize both the average level and day-to-day variability of physical activity. We experimented with two sets of activity metrics which resulted in two separate clustering analyses: one based on volume-based accelerometry data, and one based on derived intensity-based activity metrics.

For the direct accelerometry approach, input variables included mean daily steps, standard deviation (SD) of daily steps, and mean average difference (MAD) of daily steps, supplemented by equivalent statistics for distance and floors climbed. The intensity-based approach included mean, SD, and MAD of very active minutes and fairly active minutes per day, alongside activity calories and total caloric expenditure. Standard deviation and mean average difference of daily metrics were moderately to highly correlated (Spearman *ρ* range 0.79–0.92), but both were retained to capture symmetric and asymmetric variability, respectively. Wear-time (valid days) was not included as a clustering input because participants had already been filtered for ≥3 valid days and mean wear-time was 7.1 days with limited variance.

### Identification of physical activity patterns

Physical activity patterns were identified separately for the two metric sets using agglomerative hierarchical cluster analysis. Prior to clustering, all input variables were standardized to z-scores (mean 0, SD 1) to prevent metrics with larger absolute values from dominating the distance calculations. Ward's minimum variance linkage method was used to minimize within-cluster sum of squares, which tends to produce compact, well-separated clusters of approximately equal size (15). Euclidean distance was used as the dissimilarity measure. Inspection of dendrogram topologies, scree plots of fusion coefficients, and phenotypic interpretability initially identified a five-cluster architecture for both sets of metrics.

To evaluate the internal validity and stability of the initial clustering solutions, mean silhouette coefficients were calculated, and cluster stability was assessed through bootstrap resampling across 1,000 iterations. Sensitivity analyses using k-means clustering were also conducted to assess the consistency of participant assignments across alternative partitioning algorithms. Detailed validation results, including per-cluster silhouette coefficients, bootstrap Jaccard stability indices, k-means agreement metrics, and sensitivity analyses, are reported in Supplementary Tables S1–S3.

To increase statistical power for subsequent regression models without altering the underlying distance topology, the two high-volatility clusters (volume-based Clusters 2 and 5) were merged into a single consolidated “High-Variability Movers” phenotype. The *post-hoc* merge was validated by comparing it against a directly fitted four-cluster solution, reported in Supplementary Table S3. The merged approach was preferred because the direct four-cluster solution allocated only 2 participants to one cluster (volume-based), producing an uninterpretable partition. This resulted in four primary phenotypic categories for clinical comparison: Sedentary, Average Movers, Regular Exercisers, and High-Variability Movers.

### Classification agreement analysis

Agreement between the direct accelerometry phenotypes and the device-derived intensity classifications was formally evaluated across both the original 5-cluster and consolidated 4-group configurations. We computed the Adjusted Rand Index, Adjusted Mutual Information and Cramér's V to quantify partition similarity and association strength. The proportion of concordant participant assignments across the two systems was also calculated.

### Serum ¹H-NMR metabolomics profiling

Fasting serum samples were analyzed using high-throughput proton nuclear magnetic resonance (¹H-NMR) spectroscopy (Nightingale Health Plc, Helsinki, Finland). The metabolomic panel quantified 250 molecular biomarkers, providing absolute concentrations and ratio metrics across 14 lipoprotein subclasses (ranging from extremely large VLDL to small HDL), total lipid content, cholesterol subfractions, triglycerides, phospholipids, apolipoproteins (ApoA1 and ApoB), amino acids, glycolysis metabolites, ketone bodies, and the systemic inflammatory marker Glycoprotein Acetyls (GlycA).

### Clinical and imaging assessments

#### Body composition

Body composition was evaluated using the InBody 570 multi-frequency bioelectrical impedance analyzer (InBody Co., Ltd., Seoul, Korea), providing measurements of body fat percentage, skeletal muscle percentage, and muscle-to-fat ratio. Measurements were taken with participants in lightweight clothing, without shoes, following standard protocols.

#### Cardiac magnetic resonance imaging

Advanced imaging in the form of cardiac magnetic resonance imaging was performed for all participants using either the Ingenia 3.0T (Philips) or MAGNETOM Aera 1.5T (Siemens Healthineers) scanners. Variables assessed included left ventricular mass, left ventricular end diastolic volume (LVEDV), left ventricular end systolic volume (LVESV), left ventricular stroke volume (LVSV), left ventricular ejection fraction (LVEF), right ventricular end diastolic volume (RVEDV), right ventricular end systolic volume (RVESV), right ventricular stroke volume (RVSV), and right ventricular ejection fraction (RVEF). Indexes for all these variables were calculated by dividing by participants' body surface area using the Du Bois formula (16).

### Statistical analysis

Baseline characteristics across physical activity phenotypes were compared using one-way analysis of variance (ANOVA) for continuous variables and Pearson's chi-square test for categorical variables.

Prior to multivariate analysis, all 250 biomarkers were rank-based inverse normal transformed (RINT) to ensure approximate standard normality, with effect sizes reported in SD units. Sporadic missing biomarker values (<2% per analyte) were imputed to the median (0 in RINT-transformed scale) for PERMANOVA distance calculations. The proportion of variance in the 250-biomarker metabolomic profile explained by activity classification was assessed using Permutational Multivariate Analysis of Variance (PERMANOVA) on Euclidean distance matrices, with 49,999 permutations and adjustment for age, sex, and assay batch. Pairwise contrasts between phenotypes were evaluated with Holm adjustment for multiple testing. Standardized effect sizes (β in SD units) were calculated for individual biomarkers, and Benjamini-Hochberg False Discovery Rate (FDR) correction was applied across the metabolomic panel (FDR *q* < 0.05). Sensitivity analyses adjusted for smoking status and body mass index.

Associations between physical activity phenotypes and body composition or CMR parameters were evaluated using multivariable linear regression models adjusted for age and sex, with “Sedentary” serving as the reference group. Standardized regression coefficients (β, representing SD unit changes relative to “Sedentary”) and standard errors were reported. FDR correction was applied across the 16 body composition and imaging outcomes within each classification model (FDR *q* < 0.05). All statistical analyses were conducted in Stata version 17.0 (StataCorp, College Station, TX).

## Results

### Study population characteristics

The baseline characteristics of the 984 participants across volume-based phenotypes are presented in Supplementary Table S4. The cohort had a mean age of 45.6 years (SD 11.8), with 48.3% (475/984) male participants and 91.7% (902/984) ethnic Chinese representation. Significant demographic differences were observed across volume-based phenotypes, particularly for sex distribution (*P* < .001), with Sedentary individuals being predominantly female (63.0%, 271/430) and High-Variability Movers being predominantly male (61.1%, 69/113).

The mean number of valid monitoring days per participant was 7.1 days. Valid wear days were defined as days with non-missing step count data. No minimum hourly wear-time threshold was applied because only daily summary metrics were available from the data-processing pipeline. After excluding participants with fewer than three valid wear days to ensure sufficient data for intra-individual variability estimation, the final analytical dataset comprised 984 participants with complete accelerometry and clinical phenotyping, of whom 983 had complete assignments across both phenotyping models and 876 had complete serum ¹H-NMR metabolomics data. PERMANOVA analyses included 864 participants for volume-based and 873 for intensity-based phenotypes after restricting to those with both metabolomics data and complete phenotype assignments.

### Physical activity cluster characteristics

Agglomerative hierarchical clustering based on direct accelerometry data identified four consolidated activity phenotypes (Supplementary Table S4): Sedentary (*n* = 430) characterized by the lowest daily activity volume (mean 7,183 steps/day) and the lowest day-to-day variability (step MAD 2,774, step SD 1,927); Average Movers (*n* = 222) characterized by moderate daily steps (mean 8,635 steps/day, distance 6.0 km) and moderate day-to-day variability (step MAD 5,541, step SD 4,072); Regular Exercisers (*n* = 219) characterized by high daily activity volume (mean 12,089 steps/day, distance 8.6 km, 14.0 floors climbed) maintained with low day-to-day variability (step MAD 4,025, step SD 2,847); High-Variability Movers (*n* = 113, consolidating volume-based Clusters 2 and 5) characterized by the highest daily activity volume (mean 13,992 steps/day, distance 10.1 km, 18.6 floors climbed) coupled with marked day-to-day volatility (step MAD 8,618, step SD 6,327). The details of each cluster are shown in Supplementary Table S5.

Similarly, for the intensity-based solution, participants were categorized based on both the volume and the consistency of their high-intensity activity. A similar five-cluster pattern was observed (Supplementary Table S6).

Direct comparison between the volume-based and device-derived intensity classification systems revealed substantial discordance. Cross-tabulation demonstrated that the two schemes agreed poorly, yielding an Adjusted Rand Index of 0.18 for both 5-cluster and 4-group configurations, an Adjusted Mutual Information of 0.14, and a Cramér's V of 0.36 (Supplementary Table S7). Overall, only 46.2% (454/983) of participants were concordantly classified into the same phenotypic category by both methods. Major misclassifications were evident: among participants classified as Sedentary by direct accelerometry, 35.6% (153/430) were reclassified into active categories (Average Movers, Regular Exercisers, or High-Variability Movers) by the device-derived algorithm. Conversely, among participants classified as High-Variability Movers by direct accelerometry, 76.1% (86/113) were reclassified into less active categories by intensity-based metrics. Figure 1 is a Sankey diagram illustrating participant flow and reclassification between physical activity phenotypes derived from direct accelerometry metrics and proprietary device-derived intensity metrics.

**Figure 1:**
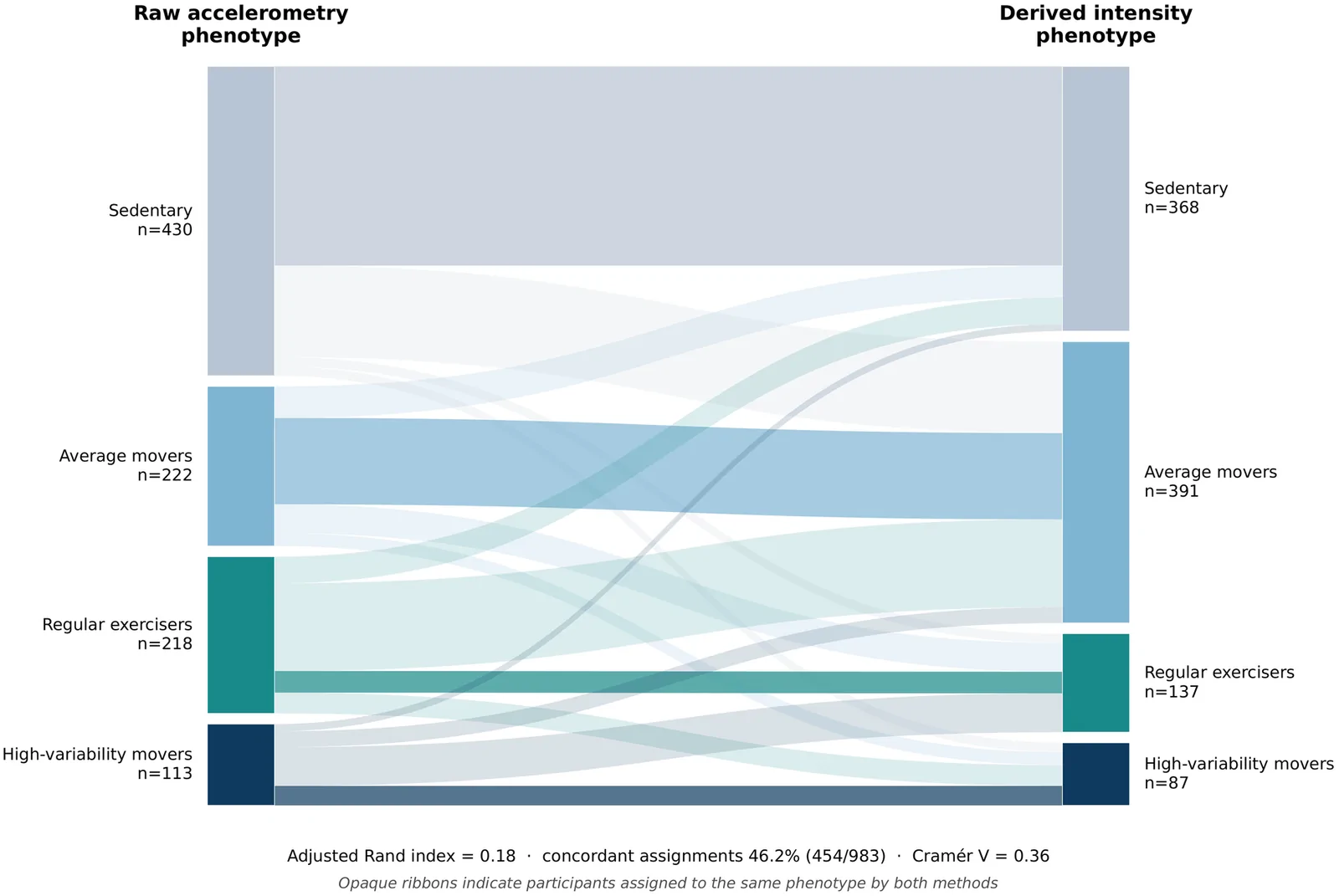
Sankey diagram illustrating participant flow and reclassification between physical activity phenotypes derived from direct accelerometry metrics (left) and proprietary device-derived intensity metrics (right).

### Metabolomic associations across classification schemes

PERMANOVA revealed that volume-based phenotypes explained a significant proportion of overall metabolomic variance (3-df omnibus R^2^ = 0.93%, *P* < .001) (Supplementary Table S8). Pairwise contrasts with Holm adjustment demonstrated distinct metabolomic separation between volume-based phenotypes. Volume-based High-Variability Movers and Regular Exercisers differed significantly from Sedentary participants (R^2^ = 1.19%, Holm *P* = .001 and R^2^ = 0.66%, Holm *P* = .018, respectively). Volume-based High-Variability Movers also differed significantly from Average Movers (R^2^ = 1.07%, Holm *P* = .045), whereas Average Movers and Regular Exercisers exhibited similar metabolomic profiles (Holm *P* = .71).

Comparing active participants (High-Variability Movers combined with Regular Exercisers) against Sedentary individuals under direct accelerometry phenotyping identified 106 out of 250 biomarkers as statistically significant at FDR *q* < 0.05 (Figure 2). Direct accelerometry phenotypes displayed favorable changes in lipoprotein profiles, marked by higher HDL subfractions (mean family *β* =  + 0.19 SD, *q* < .05), lower triglyceride-rich VLDL subfractions (mean family *β* = −0.21 SD, *q* < .05) and LDL subfractions (mean family *β* = −0.18 SD, *q* < .05), and a substantial decrease in the inflammatory marker GlycA (*β* = −0.36 SD, *q* < .05). The leading individual biomarkers included total HDL cholesterol (*β* =  + 0.343, *q* < .001), HDL phospholipids (*β* =  + 0.351, *q* < .001), medium HDL particle concentration (*β* =  + 0.351, *q* < .001), and apolipoprotein A1 (*β* =  + 0.341, *q* < .001).

**Figure 2:**
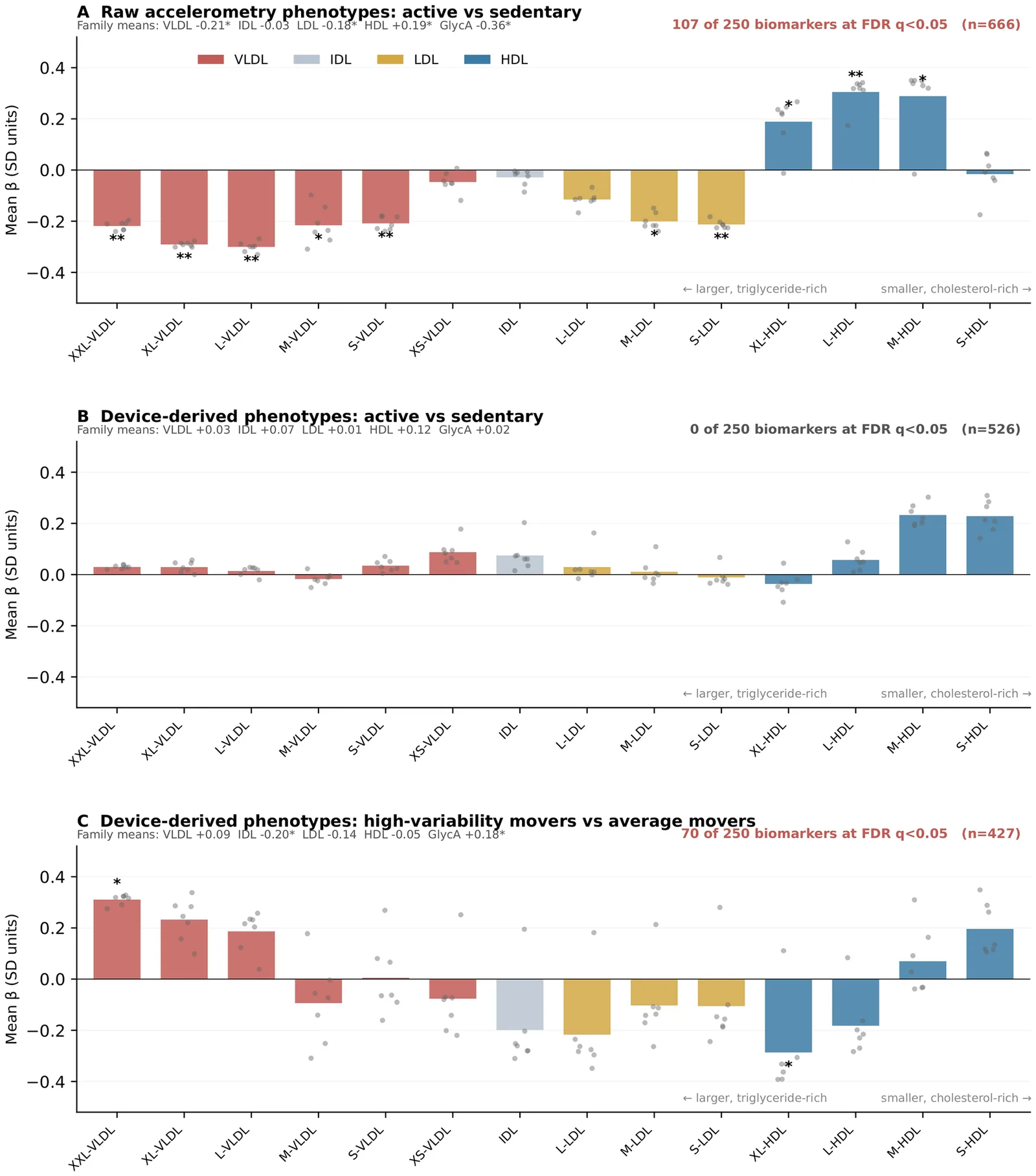
Serum ¹H-NMR metabolomic profiles across direct and device-derived physical activity classifications. Standardized differences (in standard deviation units) across 14 lipoprotein subfractions (ranging from extra-extra-large VLDL to small HDL) and the inflammatory marker GlycA. **(A)** Direct accelerometry phenotyping comparing active (regular exercisers plus high-variability movers) against sedentary participants, demonstrating favorable lipoprotein remodeling and suppressed systemic inflammation (106 of 250 biomarkers at FDR). **(B)** Device-derived intensity phenotyping comparing active against sedentary participants (PERMANOVA R^2^ = 0.23%, *P* = .29), yielding no statistically significant biomarker differences (0 of 250 biomarkers at FDR *q* < 0.05). **(C)** Device-derived intensity phenotyping comparing high-variability movers against average movers, illustrating elevated triglyceride-rich VLDL subfractions and higher GlycA inflammation (70 of 250 biomarkers at FDR). Asterisks (*) indicate statistical significance at set-level FDR.

In contrast, the association between intensity-based phenotypes and these biological profiles was less clear. Pairwise contrasts within the intensity-based classification revealed significant metabolomic separation across three comparisons involving device-derived High-Variability Movers: 83 biomarkers differed significantly vs. Regular Exercisers (R^2^ = 2.35%, Holm *P* = .005), 70 vs. Average Movers (R^2^ = 1.01%, Holm *P* = .008) and 37 vs. Sedentary participants (R^2^ = 0.89%, Holm *P* = .016) (Supplementary Table S8). However, when comparing device-derived Active participants (High-Variability Movers plus Regular Exercisers) against Sedentary individuals, PERMANOVA revealed no significant metabolomic separation (R^2^ = 0.23%, *P* = .29), and no individual biomarkers reached significance (0 of 250 at FDR *q* < 0.05). By contrast, comparing device-derived High-Variability Movers to Average Movers revealed a mixed metabolomic profile comprising 70 significant biomarkers (FDR *q* < 0.05). While this group exhibited lower IDL (*β* = −0.20 SD) and lower LDL (*β* = −0.14 SD), they concurrently exhibited elevated inflammatory GlycA (*β* =  + 0.18 SD) and increased VLDL particles (*β* =  + 0.09 SD).

In sensitivity analyses adjusting for smoking and BMI (Supplementary Table S9), omnibus associations remained statistically significant for both volume (R^2^ = 0.61%, *P* = .036) and intensity schemes (R^2^ = 0.77%, *P* = .006). Under full adjustment, active vs. sedentary profiles differed significantly for volume metrics (*P* = .005, 48 biomarkers) but not intensity metrics (*P* = .14, 10 biomarkers).

### Body composition and cardiac magnetic resonance phenotypes

Multivariable linear regression demonstrated that direct accelerometry phenotypes were significantly associated with 11 out of 16 body composition and CMR outcomes at FDR *q* < 0.05 (Supplementary Table S10). Volume-based phenotypes exhibited clear dose-response relationships consistent with exercise physiology. Relative to Sedentary individuals, volume-based High-Variability Movers displayed lower body fat percentage (*β* = −0.38 SD, *q* < .001), higher skeletal muscle percentage (*β* =  + 0.33 SD, *q* < .001), and an elevated muscle-to-fat ratio (*β* =  + 0.36 SD, *q* < .001). Furthermore, volume-based High-Variability Movers demonstrated robust, physiologically expected cardiac structural adaptation, exhibiting higher LV mass index (*β* =  + 0.35 SD, *q* = .001), LVEDV index (*β* =  + 0.50 SD, *q* < .001), LVSV index (*β* =  + 0.43 SD, *q* = .001), and RVEDV index (*β* =  + 0.51 SD, *q* < .001) without impairment of ejection fraction.

Conversely, device-derived intensity phenotypes were associated with only 6 outcomes at FDR *q* < 0.05, often demonstrating paradoxical results (Supplementary Table S10). Device-derived High-Variability Movers exhibited adverse body composition profiles relative to Sedentary individuals, including significantly higher BMI (*β* =  + 0.54 SD, *q* < .001) and waist circumference (*β* =  + 0.45 SD, *q* < .001). In terms of cardiac structure, on one hand, intensity-derived Regular Exercisers exhibited physiological cardiac remodeling, demonstrating higher LV mass index (*β* =  + 0.38 SD, *q* < .001), LVEDV index (*β* =  + 0.27 SD, *q* = .006) and RVEDV index (*β* =  + 0.31 SD, *q* = .006). However, device-derived High-Variability Movers displayed divergent cardiac structural associations, showing reduced LVEDV index (*β* = −0.18 SD, *q* = .006), reduced LVSV index (*β* = −0.28 SD, *q* = .005), and reduced RVEDV index (*β* = −0.11 SD, *q* = .006).

## Discussion

### Principal findings

This study evaluated whether physical activity phenotyping based on direct accelerometry data or device-derived intensity metrics provides closer alignment with objective circulating metabolomic biomarkers, body composition parameters, and cardiac magnetic resonance imaging. Overall, there was substantial discordance between volume-based and intensity-based activity classifications (Adjusted Rand Index = 0.18), with only 46.2% of participants assigned to the same phenotype across methods.

When evaluated against serum ¹H-NMR metabolomics, body composition, and CMR parameters, volume-based phenotypes demonstrated physiologically coherent associations. Active phenotypes defined by direct activity metrics exhibited a favorable lipoprotein remodeling pattern across 106 out of 250 measured biomarkers (FDR *q* < 0.05), characterized by higher HDL cholesterol subfractions, lower triglyceride-rich VLDL subfractions, and lower systemic GlycA inflammation. These individuals also exhibited lower body fat percentage, higher skeletal muscle mass, and larger ventricular volumes on CMR imaging. In contrast, device-derived intensity phenotypes did not identify significant metabolomic differences between active and sedentary groups in pairwise comparison (PERMANOVA *P* = .29; 0 biomarkers at FDR *q* < 0.05), despite a significant omnibus test (*P* = .003) and demonstrated inconsistent associations with body composition and cardiac structural parameters.

Both classification schemes explained under 1% of total metabolomic variance (volume-based R^2^ = 0.93%, intensity-based R^2^ = 0.80%), which is consistent with the modest effect sizes typically reported for lifestyle factors in population-based metabolomic studies. The key finding lies in the direction and biological coherence of the associations rather than in the absolute magnitude of the explained variance.

### Mechanisms of algorithmic and biological divergence

A possible explanation for these divergent associations relates to how commercial wearables estimate physical activity intensity. Many commercial devices combine wrist-worn optical photoplethysmography (PPG) heart rate data with accelerometry to estimate active minutes and energy expenditure. However, heart rate measured by PPG can be affected by factors unrelated to activity, such as psychological stress, elevated ambient temperature, caffeine intake, or autonomic variability (17, 18). As a result, increases in heart rate even without exercise may be misinterpreted by device algorithms as physical activity (19), which could lead to misclassifying less active or stressed individuals into higher intensity categories.

Furthermore, commercial wearable algorithms commonly derive activity intensity using age-predicted maximal heart rate rather than measured maximal cardiopulmonary capacity (20). Given the substantial individual variation in maximal heart rate, this may misclassify activity intensity, overestimating vigorous activity in some individuals while underestimating it in highly trained individuals.

This potential misclassification could help explain why participants categorized as “high-variability movers” by device-derived metrics exhibited higher body mass index, larger waist circumferences, elevated GlycA levels, and smaller ventricular volumes. By contrast, direct accelerometry metrics, such as daily steps and distance, reflect movement volume directly without relying on proprietary intensity thresholding. In our analyses, volume-based phenotypes consistently aligned with expected physiological conditioning across circulating metabolomic, body composition, and cardiac imaging domains.

At the same time, alternative explanations for the observed classification discordance and divergent biological associations should also be considered. Although both sets of metrics were evaluated using the same agglomerative hierarchical clustering approach, minimizing the likelihood that algorithmic artifacts caused the divergence, unsupervised clustering methods are inherently sensitive to the variables included and their underlying variance. Furthermore, residual confounding from unmeasured lifestyle factors, such as dietary patterns, sleep, or acute psychological stress, may impact heart-rate-derived intensity metrics more than direct accelerometry counts.

### Implications for digital health research

This study has significant implications for clinical research utilizing wearable data. Given that volume-based activity metrics had more physiologically consistent associations with biological markers than metrics derived from proprietary algorithms, digital health research should prioritize volume-based accelerometry data. Relying on manufacturer-derived values introduces an additional layer of bias that can lead to erroneous conclusions about the efficacy of physical activity interventions (21).

For clinicians and researchers, prioritizing direct accelerometry data, including steps, floors climbed, and distance, could improve reproducibility across different device brands and provide a more robust link to physiological outcomes. This approach also enhances transparency, as direct activity metrics can be interpreted directly without requiring knowledge of proprietary algorithms. As digital health tools increasingly inform clinical decision-making and health policy (22), the validity and equity of underlying data are paramount.

### Limitations

This study has several limitations. First, the cross-sectional design prevents us from establishing causal relationships between activity patterns, metabolomic profiles, body composition, and cardiac remodeling. Longitudinal studies incorporating serial serum metabolomic profiling are required to achieve that purpose. Second, the use of a single device (Fitbit Charge HR) means the derived intensity-based results are specific to one manufacturer's algorithms and may not generalize to other wearable brands. However, this limitation strengthens rather than weakens our core argument for using raw data, as direct activity metrics are more likely to be comparable across devices. Third, the absence of a minimum daily wear-time criterion may introduce measurement error, as even partial-wear days where the device was removed for several hours would be counted as valid and could inflate day-to-day variability metrics. Fourth, wrist-worn accelerometers may not fully capture all forms of physical activity. Activities such as cycling, swimming, and resistance training are often poorly recorded or do not contribute to step counts. Individuals who engage in these forms of exercise may therefore have been classified as less active, potentially weakening the observed physiological and metabolomic associations.

Furthermore, our study population was predominantly Chinese and relatively healthy, which may limit generalizability to other ethnic groups or clinical populations. The lack of ethnic diversity also prevented us from examining potential racial disparities in sensor accuracy, which was documented in previous research (10). In addition, we did not have access to the proprietary algorithms underlying the derived metrics, preventing us from understanding the specific sources of misclassification. CMR imaging was performed using two scanner platforms (3.0T Philips and 1.5T Siemens), and while all measurements were indexed to body surface area, we did not include scanner type as a covariate in CMR analyses. However, prior work has shown that indexed CMR values are comparable across field strengths in healthy populations (23). Finally, while our sample size was adequate for cluster analysis and regression modeling, larger studies would provide more precise estimates and enable examination of effect modification by demographic factors.

### Future directions

Future research should compare direct vs. derived metrics across multiple device brands and in more diverse populations to establish the generalizability of our findings. Validation studies using criterion measures (e.g., doubly labeled water for energy expenditure, direct observation for activity type) would help elucidate the specific mechanisms of bias in derived metrics. Additionally, longitudinal studies examining whether volume-based activity phenotypes predict incident cardiovascular events and mortality would establish their clinical utility for risk stratification.

From a methodological perspective, development of standardized, open-source algorithms for deriving activity intensity from direct accelerometry could address concerns about proprietary “black boxes” while still providing researchers with interpretable intensity metrics. Such efforts would require collaboration between device manufacturers, academic researchers, and regulatory bodies to establish consensus standards.

## Conclusion

While device-derived intensity metrics remain widely used, our findings demonstrate that reliance on proprietary algorithms introduces substantial classification discordance and may generate paradoxical health associations. In contrast, activity phenotypes derived from direct accelerometry metrics provide a transparent, reproducible, and physiologically coherent foundation for digital phenotyping, capturing physical activity adaptations across circulating metabolomics, body composition, and cardiovascular structure.

## Data Availability

The datasets analyzed during the current study cannot be shared publicly due to the need to protect the privacy of individuals that participated in the study, but are available from the corresponding author on reasonable request. Requests to access the datasets should be directed to Weiting Huang, huang.weiting@singhealth.com.sg.
