# Supplementary material for: Physical activity phenotypes from volume-based versus intensity-based wearable metrics: discordant classification and divergent metabolomic, body composition, and cardiac magnetic resonance associations
Source: Front Digit Health. 2026 Oct 26;8:1982891. doi: 10.3389/fdgth.2026.1982891 (PMC13745681; doi:10.3389/fdgth.2026.1982891)
Supplement: Supplementary file 1 [file Table1.docx]

**Supplementary Tables**

**Supplementary Table 1. Cluster Validation Metrics**

| **Metric** | **Volume-based** | **Intensity-based** |
| --- | --- | --- |
| **Overall silhouette (5 clusters)** | 0.27 | 0.20 |
| **Silhouette: Sedentary** | 0.41 | 0.33 |
| **Silhouette: Average Movers** | 0.24 | 0.13 |
| **Silhouette: Regular Exercisers** | 0.11 | 0.11 |
| **Silhouette: Cluster 2 (n=17)** | 0.01 | 0.09 |
| **Silhouette: Cluster 5** | 0.09 | 0.06 |
| **K-means agreement (ARI / AMI)** | 0.67 / 0.64 | 0.81 / 0.82 |
| **Bootstrap Jaccard stability: Sedentary** | 0.90 | 0.86 |
| **Bootstrap Jaccard stability: Average Movers** | 0.53 | 0.78 |
| **Bootstrap Jaccard stability: Regular Exercisers** | 0.50 | 0.71 |
| **Bootstrap Jaccard stability: Cluster 2 (n=17)** | 0.40 | 0.74 |
| **Bootstrap Jaccard stability: Cluster 5** | 0.74 | 0.74 |

*Jaccard stability: mean Jaccard similarity of each cluster across 1,000 bootstrap resamples. Values >0.75 indicate highly stable clusters; 0.50–0.75 moderate; <0.50 unstable (Hennig, 2007).*

**Supplementary Table 2. Spearman Intercorrelations Among Clustering Input Variables (SD–MAD pairs)**

| **Metric triple** | **SD–MAD ρ** | **Mean–SD ρ** | **Mean–MAD ρ** |
| --- | --- | --- | --- |
| **Volume-based inputs** |  |  |  |
| **Steps/day** | 0.79 | 0.42 | 0.44 |
| **Distance/day** | 0.81 | 0.43 | 0.49 |
| **Floors/day** | 0.88 | 0.71 | 0.80 |
| **Intensity-based inputs** |  |  |  |
| **Very-active min/day** | 0.92 | 0.84 | 0.93 |
| **Fairly-active min/day** | 0.90 | 0.80 | 0.91 |

**Supplementary Table 3. Direct 4-Cluster vs Merged 4-Group Comparison**

| **Metric** | **Volume-based** | **Intensity-based** |
| --- | --- | --- |
| **Direct 4-cluster sizes** | 472, 394, 116, 2 | 405, 391, 166, 31 |
| **Direct 4-cluster silhouette** | 0.28 | 0.20 |
| **ARI (direct 4 vs merged 4)** | 0.68 | 0.85 |
| **AMI (direct 4 vs merged 4)** | 0.67 | 0.79 |
| **Winsorization (99th pct) ARI vs published 5-cluster** | 0.89 | 0.61 |

*ARI, Adjusted Rand Index; AMI, Adjusted Mutual Information*

**Supplementary Table 4. Participant characteristics by volume-based accelerometry phenotype**

| Characteristic | Overall | Sedentary | Average movers | Regular exercisers | High-variability movers | P value |
| --- | --- | --- | --- | --- | --- | --- |
| n | 984 | 430 | 222 | 219 | 113 |  |
| Demographics |  |  |  |  |  |  |
| Age, years | 45.6 (11.8) | 45.9 (11.2) | 44.1 (12.4) | 46.0 (11.7) | 46.7 (13.1) | .161 |
| Male sex, n (%) | 475 (48.3) | 159 (37.0)^b, c, d^ | 126 (56.8)^a^ | 121 (55.3)^a^ | 69 (61.1)^a^ | <.001 |
| Chinese ethnicity, n (%) | 902 (91.7) | 399 (92.8) | 195 (87.8) | 205 (93.6) | 103 (91.2) | .107 |
| Ever smoker, n (%) | 303 (30.8) | 148 (34.4) | 66 (29.7) | 58 (26.5) | 31 (27.4) | .153 |
| Wearable activity metrics |  |  |  |  |  |  |
| Mean daily steps | 9384 (3251) | 7183 (1800) ^b, c, d^ | 8635 (1667) ^a, c, d^ | 12089 (1877) ^a, b, d^ | 13992 (3543) ^a, b, c^ | <.001 |
| Mean daily distance, km | 6.6 (2.5) | 4.9 (1.3) ^b, c, d^ | 6.0 (1.2) ^a, c, d^ | 8.6 (1.5) ^a, b, d^ | 10.1 (2.7) ^a, b, c^ | <.001 |
| Mean daily floors climbed | 9.4 (11.0) | 5.6 (3.7) ^b, c, d^ | 7.5 (4.5) ^a, c, d^ | 14.0 (9.8) ^a, b, d^ | 18.6 (24.6) ^a, b, c^ | <.001 |
| Daily step variability (SD) | 3121 (1827) | 1927 (742) ^b, c, d^ | 4072 (1106) ^a, c, d^ | 2847 (931) ^a, b, d^ | 6327 (2305) ^a, b, c^ | <.001 |
| Daily step variability (MAD) | 4348 (2379) | 2774 (986) ^b, c, d^ | 5541 (1354) ^a, c, d^ | 4025 (1318) ^a, b, d^ | 8618 (2829) ^a, b, c^ | <.001 |
| Very-active minutes/day | 18.0 (19.5) | 10.0 (12.6) ^b, c, d^ | 16.9 (13.9) ^a, c, d^ | 25.2 (20.4) ^a, b, d^ | 36.8 (29.1) ^a, b, c^ | <.001 |
| Fairly-active minutes/day | 17.5 (18.4) | 11.6 (13.1) ^b, c, d^ | 17.0 (14.9) ^a, c, d^ | 24.8 (23.1) ^a, b, d^ | 26.4 (22.7) ^a, b, c^ | <.001 |
| Activity calories/day | 857 (443) | 700 (350) ^b, c, d^ | 822 (371) ^a, c, d^ | 1066 (483) ^a, b, d^ | 1121 (526) ^a, b, c^ | <.001 |
| Total calories/day | 2171 (510) | 1987 (456) ^b, c, d^ | 2175 (435) ^a, c, d^ | 2397 (540) ^a, b, d^ | 2428 (504) ^a, b, c^ | <.001 |
| Body composition |  |  |  |  |  |  |
| Body mass index, kg/m² | 23.5 (3.7) | 23.8 (4.0) | 23.5 (3.3) | 23.3 (3.6) | 22.9 (2.9) | .116 |
| Waist circumference, cm | 83.0 (11.0) | 83.3 (11.2) | 83.2 (11.1) | 82.5 (11.0) | 82.3 (9.6) | .731 |
| Waist-hip ratio | 0.88 (0.08) | 0.87 (0.08) | 0.88 (0.08) | 0.88 (0.07) | 0.88 (0.06) | .888 |
| Body fat, % | 28.7 (8.5) | 30.9 (8.3)^b, c, d^ | 27.8 (8.2)^a, d^ | 27.0 (8.5)^a^ | 25.5 (8.3)^a, b^ | <.001 |
| Skeletal muscle, % | 38.9 (5.4) | 37.6 (5.0) | 39.6 (5.2) | 39.8 (5.5) | 40.9 (5.5) | <.001 |
| Cardiometabolic measures |  |  |  |  |  |  |
| Total cholesterol, mmol/L | 5.36 (0.95) | 5.41 (0.94) | 5.31 (1.01) | 5.31 (0.95) | 5.34 (0.90) | .530 |
| LDL cholesterol, mmol/L | 3.34 (0.83) | 3.37 (0.81) | 3.35 (0.87) | 3.30 (0.82) | 3.26 (0.83) | .537 |
| HDL cholesterol, mmol/L | 1.50 (0.34) | 1.49 (0.34)^d^ | 1.44 (0.33)^d^ | 1.53 (0.34) | 1.59 (0.33)^a, b^ | <.001 |
| Triglycerides, mmol/L | 1.13 (0.66) | 1.18 (0.64) | 1.18 (0.74) | 1.05 (0.56) | 1.02 (0.70) | .015 |
| Fasting glucose, mmol/L | 5.23 (0.57) | 5.26 (0.64) | 5.22 (0.57) | 5.19 (0.49) | 5.23 (0.47) | .459 |
| Cardiac magnetic resonance |  |  |  |  |  |  |
| LV mass index, g/m² | 44.4 (9.2) | 42.6 (8.6)^b, c, d^ | 44.9 (9.0)^a, d^ | 45.4 (9.2)^a, d^ | 48.2 (10.4)^a, b, c^ | <.001 |
| LV end-diastolic volume index, mL/m² | 72.6 (10.8) | 70.4 (10.1) ^b, c, d^ | 73.8 (10.0) ^a, d^ | 73.5 (10.6) | 77.0 (13.0) ^a, b, c^ | <.001 |
| LV ejection fraction, % | 60.9 (5.8) | 61.7 (5.9)^b, c^ | 60.0 (5.7)^a^ | 60.5 (5.5)^a^ | 60.4 (5.6) | <.001 |
| LV stroke volume index, mL/m² | 44.0 (6.2) | 43.2 (6.2) | 44.0 (5.6) | 44.2 (6.2) | 46.2 (6.9) | <.001 |
| RV end-diastolic volume index, mL/m² | 77.0 (13.5) | 73.9 (13.1) ^b, c, d^ | 78.1 (12.8) ^a, d^ | 78.8 (12.7) | 82.9 (15.1) ^a, b, c^ | <.001 |
| RV ejection fraction, % | 57.1 (7.1) | 57.9 (6.8) | 56.0 (7.9) | 56.6 (6.8) | 57.1 (6.9) | .007 |
| Metabolomic subsample |  |  |  |  |  |  |
| NMR metabolomics available, n (%) | 864 (87.8) | 374 (87.0) | 198 (89.2) | 197 (90.0) | 95 (84.1) | .378 |

*Values are mean (SD) unless stated. High-variability movers combine volume-based clusters 2 and 5. P values from one-way analysis of variance for continuous variables and the chi-square test for categorical variables, comparing the four phenotypes. MAD, mean average difference; LV, left ventricular; RV, right ventricular. For daily variability metrics (i.e. Daily step variability [SD] and [MAD]), values represent the between-participant mean of each individual's day-to-day within-person variability across the monitoring period. Post-hoc pairwise comparisons were conducted with Holm-adjusted P-values, and superscript letters indicate statistically significant pairwise differences: ᵃ = differs from Sedentary, ᵇ = differs from Average Movers, ᶜ = differs from Regular Exercisers, ᵈ = differs from High-Variability Movers.*

**Supplementary Table 5. Volume-Based Accelerometry Activity Clusters**

| Fitbit metric | Cluster 1 (Regular Exercisers) (n=219) | Cluster 2 (High-Variability Movers, elevation gain activities) (n=17) | Cluster 3 (Average Movers) (n=222) | Cluster 4 (Sedentary) (n=430) | Cluster 5 (High-Variability Movers) (n=96) |
| --- | --- | --- | --- | --- | --- |
| Steps/day | 12088.81 (1877.42) | 14162.18 (3387.77) | 8635.09 (1666.81) | 7183.27 (1800.43) | 13961.58 (3585.74) |
| Mean average difference of daily steps | 4025.29 (1318.47) | 8265.94 (2953.93) | 5541.18 (1353.67) | 2774.07 (985.87) | 8680.71 (2817.29) |
| Standard Deviation of difference of daily steps | 2847.13 (930.80) | 6172.64 (3322.11) | 4072.09 (1106.25) | 1926.76 (742.31) | 6353.83 (2098.06) |
| Floors/day | 13.97 (9.76) | 57.80 (44.59) | 7.51 (4.49) | 5.62 (3.71) | 11.62 (7.33) |
| Mean average difference of daily floors | 10.20 (8.25) | 70.36 (58.03) | 7.04 (4.68) | 4.46 (3.40) | 11.26 (9.45) |
| Standard Deviation of difference of daily floors | 8.57 (9.40) | 71.77 (60.62) | 5.50 (4.58) | 3.55 (3.27) | 8.48 (7.71) |
| Distance/day, km | 8.56 (1.52) | 9.78 (2.47) | 6.03 (1.22) | 4.90 (1.32) | 10.16 (2.72) |
| Mean average difference of daily distance | 2.98 (0.96) | 5.84 (2.13) | 3.94 (0.94) | 1.93 (0.68) | 6.71 (2.34) |
| Standard Deviation of difference of daily distance | 2.12 (0.74) | 4.42 (2.59) | 2.93 (0.76) | 1.39 (0.55) | 5.10 (1.89) |

*Values are mean (SD). Agglomerative hierarchical clustering (Ward linkage, Euclidean distance) on nine z-standardized direct accelerometry metrics. Clusters 2 and 5 were combined into a single High-Variability Movers phenotype (n=113) for the main analyses.*

**Supplementary Table 6. Intensity-Based Derived Metrics Clusters**

| **Fitbit metric** | **Cluster 1 (Regular Exercisers) (n=137)** | **Cluster 2 (High Intensity High-Variability Movers) (n=17)** | **Cluster 3 (Average Movers) (n=391)** | **Cluster 4 (Sedentary) (n=368)** | **Cluster 5 (High-Variability Movers) (n=70)** |
| --- | --- | --- | --- | --- | --- |
| **Minutes very active/day, min** | 33.63 (16.84) | 71.71 (29.02) | 17.43 (10.97) | 5.06 (5.82) | 45.47 (31.07) |
| **Mean average difference of daily minutes very active** | 41.68 (16.74) | 99.44 (29.67) | 19.16 (10.85) | 6.02 (6.45) | 41.61 (21.57) |
| **Standard Deviation of difference of daily minutes very active** | 38.02 (20.17) | 91.66 (46.10) | 16.71 (11.07) | 6.30 (6.81) | 32.04 (20.14) |
| **Minutes fairly active/day, min** | 21.60 (9.51) | 62.08 (45.26) | 17.87 (10.57) | 5.80 (5.24) | 57.58 (22.43) |
| **Mean average difference of daily minutes fairly active** | 23.27 (9.17) | 69.53 (41.32) | 18.84 (10.21) | 6.67 (5.42) | 57.95 (19.43) |
| **Standard Deviation of difference of daily minutes fairly active** | 18.91 (9.90) | 55.95 (34.06) | 15.82 (10.43) | 6.49 (5.44) | 48.42 (22.30) |
| **Activity calories/day** | 1213.68 (329.08) | 1835.82 (740.57) | 913.88 (240.17) | 506.93 (294.72) | 1445.44 (393.75) |
| **Mean average difference of daily activity calories** | 750.33 (210.31) | 1513.34 (403.68) | 455.74 (137.51) | 237.21 (134.19) | 796.46 (216.57) |
| **Standard Deviation of difference of daily activity calories** | 579.20 (187.83) | 1292.90 (366.00) | 371.43 (133.52) | 214.17 (129.76) | 591.93 (231.72) |
| **Total calories burned/day** | 2641.31 (422.10) | 3225.67 (725.75) | 2169.50 (375.61) | 1854.96 (360.79) | 2666.58 (502.04) |
| **Mean average difference of total daily calories burned** | 705.18 (220.67) | 1407.59 (322.14) | 420.39 (135.62) | 293.95 (133.76) | 715.24 (209.19) |
| **Standard Deviation of difference of total daily calories burned** | 529.90 (198.73) | 1181.55 (373.85) | 357.60 (155.70) | 252.02 (130.52) | 536.12 (231.51) |

*Values are mean (SD). Agglomerative hierarchical clustering (Ward linkage, Euclidean distance) on twelve z-standardized intensity-based metrics. Clusters 2 and 5 were combined into a single High-Variability Movers phenotype (n=87) for subsequent analyses.*

**Supplementary Table 7. Agreement between the volume-based and intensity-based classifications**

**Panel A. Five-cluster cross-tabulation**

| Volume-Based cluster | Intensity-Based 1 | Intensity-Based 2 | Intensity-Based 3 | Intensity-Based 4 | Intensity-Based 5 |
| --- | --- | --- | --- | --- | --- |
| Cluster 1 | 30 | 3 | 122 | 37 | 26 |
| Cluster 2 | 6 | 2 | 6 | 2 | 1 |
| Cluster 3 | 40 | 4 | 120 | 44 | 14 |
| Cluster 4 | 13 | 4 | 127 | 277 | 9 |
| Cluster 5 | 48 | 4 | 16 | 8 | 20 |

**Panel B. Four-group cross-tabulation, high-variability movers combined**

| Volume-Based phenotype | Intensity-Based: sedentary | Intensity-Based: average movers | Intensity-Based: regular exercisers | Intensity-Based: high-variability movers | Row total |
| --- | --- | --- | --- | --- | --- |
| Sedentary | 277 | 127 | 13 | 13 | 430 |
| Average movers | 44 | 120 | 40 | 18 | 222 |
| Regular exercisers | 37 | 122 | 30 | 29 | 218 |
| High-variability movers | 10 | 22 | 54 | 27 | 113 |

**Panel C. Agreement statistics**

| Statistic | Value | Interpretation |
| --- | --- | --- |
| Adjusted Rand index, 5 clusters | 0.18 | 0 = chance agreement, 1 = identical partitions |
| Adjusted Rand index, 4 groups | 0.18 | 0 = chance agreement, 1 = identical partitions |
| Adjusted mutual information, 5 clusters | 0.14 | Shared information between partitions |
| Adjusted mutual information, 4 groups | 0.15 | Shared information between partitions |
| Cramér V, 5 clusters | 0.32 | Strength of association between the two classifications |
| Cramér V, 4 groups | 0.36 | Strength of association between the two classifications |
| Concordant assignments | 46.2% (454/983) | Participants placed in the same phenotype by both methods |

*Shaded cells in Panel B indicate concordant assignments. Analyses include the 983 participants with a phenotype assignment under both classifications. One participant with a volume-based regular-exerciser assignment lacked a device-derived assignment, hence the regular exercisers row totals 218 versus 219 in Supplementary Table 4.*

**Supplementary Table 8. Metabolomic associations of the two classifications (250 NMR biomarkers)**

**Panel A. Total metabolomic variance explained by phenotype (Omnibus PERMANOVA)**

| Classification | df | Partial R², % | P value |
| --- | --- | --- | --- |
| Volume-based accelerometry phenotypes |  |  |  |
| Four groups (high-variability movers combined) | 3 | 0.93 | <.001 |
| Five clusters (uncombined) | 4 | 0.98 | .004 |
| Intensity-based device-derived phenotypes |  |  |  |
| Four groups (high-variability movers combined) | 3 | 0.80 | .003 |
| Five clusters (uncombined) | 4 | 0.86 | .012 |

**Panel B. Pairwise contrasts between phenotypes**

| Contrast | n vs n | Partial R², % | P value | Holm-adjusted P | Biomarkers at FDR q<0.05 |
| --- | --- | --- | --- | --- | --- |
| Volume-based phenotypes — omnibus 3 df: R² 0.93%, P <.001 |  |  |  |  |  |
| High-variability movers vs Sedentary | 95 vs 374 | 1.19 | <.001 | .001 | 91 |
| Regular exercisers vs Sedentary | 197 vs 374 | 0.66 | .004 | .018 | 62 |
| High-variability movers vs Average movers | 95 vs 198 | 1.07 | .011 | .045 | 29 |
| High-variability movers vs Regular exercisers | 95 vs 197 | 0.44 | .238 | .713 | 0 |
| Regular exercisers vs Average movers | 197 vs 198 | 0.29 | .295 | .713 | 0 |
| Average movers vs Sedentary | 198 vs 374 | 0.14 | .530 | .713 | 0 |
| Active (HVM + RE) vs Sedentary | 292 vs 374 | 0.94 | <.001 | — | 106 |
| Intensity-based phenotypes — omnibus 3 df: R² 0.80%, P .003 |  |  |  |  |  |
| High-variability movers vs Regular exercisers | 80 vs 121 | 2.35 | <.001 | .005 | 83 |
| High-variability movers vs Average movers | 80 vs 347 | 1.01 | .002 | .008 | 70 |
| High-variability movers vs Sedentary | 80 vs 325 | 0.89 | .004 | .016 | 37 |
| Regular exercisers vs Sedentary | 121 vs 325 | 0.27 | .266 | .799 | 0 |
| Average movers vs Sedentary | 347 vs 325 | 0.15 | .376 | .799 | 0 |
| Regular exercisers vs Average movers | 121 vs 347 | 0.06 | .965 | .965 | 0 |
| Active (HVM + RE) vs Sedentary | 201 vs 325 | 0.23 | .287 | — | 0 |

**Panel C. Lipoprotein family and inflammation effects, mean β (SD units)**

| Contrast | VLDL | IDL | LDL | HDL | GlycA |
| --- | --- | --- | --- | --- | --- |
| Volume-based: active vs sedentary | −0.21* | −0.03 | −0.18* | +0.19* | −0.36* |
| Intensity-based: active vs sedentary | +0.03 | +0.07 | +0.01 | +0.12 | +0.02 |
| Intensity-based: high-variability movers vs average movers | +0.09 | −0.20 | −0.14 | −0.05 | +0.18 |

**Panel D. Leading biomarkers, volume-based accelerometry phenotypes, active versus sedentary**

| Biomarker | Description | β (SD units) | SE | FDR q |
| --- | --- | --- | --- | --- |
| HDL-C | HDL cholesterol | +0.343 | 0.072 | .0001 |
| HDL-PL | Phospholipids in HDL | +0.351 | 0.072 | .0001 |
| HDL-CE | Cholesteryl esters in HDL | +0.344 | 0.072 | .0001 |
| HDL-L | Total lipids in HDL | +0.350 | 0.072 | .0001 |
| L-HDL-L | Total lipids in large HDL | +0.338 | 0.071 | .0001 |
| L-HDL-PL | Phospholipids in large HDL | +0.342 | 0.071 | .0001 |
| M-HDL-P | Concentration of medium HDL particles | +0.351 | 0.073 | .0001 |
| M-HDL-C | Cholesterol in medium HDL | +0.349 | 0.073 | .0001 |
| M-HDL-CE | Cholesteryl esters in medium HDL | +0.351 | 0.074 | .0001 |
| XL-HDL-FC % | Free cholesterol to total lipids ratio in very large HDL | −0.340 | 0.071 | .0001 |
| ApoA1 | Apolipoprotein A1 | +0.341 | 0.072 | .0001 |
| L-HDL-P | Concentration of large HDL particles | +0.334 | 0.071 | .0001 |

*Metabolomic data were available for 876 participants, of whom 864 had a direct accelerometry phenotype and 873 a device-derived phenotype. Sample sizes for each specific comparison are listed in Panel B. Partial R² from permutational multivariate analysis of variance on Euclidean distances, adjusted for age, sex and assay batch, using 49,999 permutations. Holm adjustment applied across the six pairwise contrasts within each classification. High-variability movers combine clusters 2 and 5 within each classification. Active is defined as high-variability movers plus regular exercisers combined. The Active vs Sedentary contrast is not part of the pairwise set and therefore not Holm-adjusted. The values in Panel C represent the average standardized effect across all biomarkers within each lipoprotein class; * denotes FDR q<0.05 at set level.*

**Supplementary Table 9. Sensitivity Analyses: PERMANOVA with Additional Covariates**

| **Classification** | **Adjustment** | **n** | **Partial R², %** | **P value** |
| --- | --- | --- | --- | --- |
| **Volume-based (omnibus)** | Age, sex, batch | 863 | 0.93 | <.001 |
|  | + Smoking | 863 | 0.85 | .003 |
|  | + BMI | 863 | 0.63 | .031 |
|  | + Smoking + BMI | 863 | 0.61 | .036 |
| **Intensity-based (omnibus)** | Age, sex, batch | 872 | 0.80 | .004 |
|  | + Smoking | 872 | 0.79 | .005 |
|  | + BMI | 872 | 0.79 | .005 |
|  | + Smoking + BMI | 872 | 0.77 | .006 |

*Smoking was defined as ever having smoked (30.8% of cohort). BMI was analyzed as a continuous variable. The sensitivity analysis restricts to participants with non-missing smoking and BMI data (n = 863 vs 864 for volume-based; n = 872 vs 873 for intensity-based).*

**Supplementary Table 10. Body composition and cardiac magnetic resonance phenotypes by classification**

| Outcome | Volume-Based: Average Movers | Volume-Based: Regular Exercisers | Volume-Based: High-Variability Movers | Volume-Based q | Intensity-Based: Average Movers | Intensity-Based: Regular Exercisers | Intensity-Based: High-Variability Movers | Intensity-Based q |
| --- | --- | --- | --- | --- | --- | --- | --- | --- |
| Body composition |  |  |  |  |  |  |  |  |
| Body mass index | −0.15 | −0.19 | −0.34 | .008 | +0.13 | +0.47 | +0.54 | <.001 |
| Waist circumference | −0.14 | −0.20 | −0.27 | .016 | +0.08 | +0.35 | +0.45 | <.001 |
| Waist-hip ratio | −0.12 | −0.12 | −0.11 | .336 | −0.01 | +0.06 | +0.12 | .695 |
| Body fat, % | −0.13 | −0.27 | −0.38 | <.001 | −0.03 | +0.11 | +0.18 | .182 |
| Skeletal muscle, % | +0.11 | +0.20 | +0.33 | <.001 | +0.05 | −0.06 | −0.12 | .266 |
| Muscle-to-fat ratio | +0.15 | +0.25 | +0.36 | <.001 | +0.10 | +0.11 | −0.05 | .294 |
| Cardiac MRI |  |  |  |  |  |  |  |  |
| LV mass index | +0.01 | +0.10 | +0.35 | .001 | −0.01 | +0.38 | +0.14 | <.001 |
| LV end-diastolic volume index | +0.16 | +0.19 | +0.50 | <.001 | +0.02 | +0.27 | −0.18 | .006 |
| LV end-systolic volume index | +0.20 | +0.19 | +0.39 | <.001 | +0.06 | +0.20 | −0.03 | .220 |
| LV ejection fraction | −0.17 | −0.12 | −0.12 | .187 | −0.08 | −0.06 | −0.13 | .695 |
| LV stroke volume index | +0.05 | +0.12 | +0.43 | .001 | −0.03 | +0.24 | −0.28 | .005 |
| LV cardiac index | +0.06 | +0.02 | +0.05 | .883 | +0.05 | +0.24 | +0.18 | .194 |
| RV end-diastolic volume index | +0.13 | +0.23 | +0.51 | <.001 | +0.04 | +0.31 | −0.11 | .006 |
| RV end-systolic volume index | +0.15 | +0.18 | +0.29 | .006 | +0.06 | +0.23 | −0.00 | .182 |
| RV ejection fraction | −0.10 | −0.06 | +0.06 | .473 | −0.06 | −0.08 | −0.10 | .710 |
| Ascending aorta index | −0.13 | −0.02 | −0.01 | .527 | −0.09 | −0.04 | −0.13 | .695 |

*Values are adjusted differences in SD units relative to the sedentary phenotype, from linear regression adjusted for age and sex. FDR q values are corrected across the 16 outcomes within each classification. Volume-based phenotypes were associated with 11 of 16 outcomes and intensity-based phenotypes with 6. Shaded cells highlight high-variability mover effects of opposite sign between the two classifications. Cardiac measures are indexed to body surface area. Analytic n ranged from 886 to 980 (volume-based) and 894 to 984 (intensity-based) depending on outcome availability. LV, left ventricular; RV, right ventricular.*
